# BpWrapper: BioPerl-based sequence and tree utilities for rapid prototyping of bioinformatics pipelines

**DOI:** 10.1186/s12859-018-2074-9

**Published:** 2018-03-02

**Authors:** Yözen Hernández, Rocky Bernstein, Pedro Pagan, Levy Vargas, William McCaig, Girish Ramrattan, Saymon Akther, Amanda Larracuente, Lia Di, Filipe G. Vieira, Wei-Gang Qiu

**Affiliations:** 1 Department of Biological Sciences, Hunter College, City University of New York, New York, 10065 USA; 2 Graduate Center, City University of New York, New York, 10016 USA; 3 Graduate Program in Bioinformatics, Boston University, Boston, MA 02215 USA; 4 Centre for GeoGenetics, Natural History Museum of Denmark, University of Copenhagen, Copenhagen, Denmark; 5 Department of Physiology and Biophysics & Institute for Computational Biomedicine, Weil Cornell Medical College, New York, NY 10021 USA

**Keywords:** FASTA sequences, NEWICK tree, Sequence alignments, UNIX utilities, BioPerl

## Abstract

**Background:**

Automated bioinformatics workflows are more robust, easier to maintain, and results more reproducible when built with command-line utilities than with custom-coded scripts. Command-line utilities further benefit by relieving bioinformatics developers to learn the use of, or to interact directly with, biological software libraries. There is however a lack of command-line utilities that leverage popular Open Source biological software toolkits such as BioPerl (http://bioperl.org) to make many of the well-designed, robust, and routinely used biological classes available for a wider base of end users.

**Results:**

Designed as standard utilities for UNIX-family operating systems, BpWrapper makes functionality of some of the most popular BioPerl modules readily accessible on the command line to novice as well as to experienced bioinformatics practitioners. The initial release of BpWrapper includes four utilities with concise command-line user interfaces, bioseq, bioaln, biotree, and biopop, specialized for manipulation of molecular sequences, sequence alignments, phylogenetic trees, and DNA polymorphisms, respectively. Over a hundred methods are currently available as command-line options and new methods are easily incorporated. Performance of BpWrapper utilities lags that of precompiled utilities while equivalent to that of other utilities based on BioPerl. BpWrapper has been tested on BioPerl Release 1.6, Perl versions 5.10.1 to 5.25.10, and operating systems including Apple macOS, Microsoft Windows, and GNU/Linux. Release code is available from the Comprehensive Perl Archive Network (CPAN) at https://metacpan.org/pod/Bio::BPWrapper. Source code is available on GitHub at https://github.com/bioperl/p5-bpwrapper.

**Conclusions:**

BpWrapper improves on existing sequence utilities by following the design principles of Unix text utilities such including a concise user interface, extensive command-line options, and standard input/output for serialized operations. Further, dozens of novel methods for manipulation of sequences, alignments, and phylogenetic trees, unavailable in existing utilities (e.g., EMBOSS, Newick Utilities, and FAST), are provided. Bioinformaticians should find BpWrapper useful for rapid prototyping of workflows on the command-line without creating custom scripts for comparative genomics and other bioinformatics applications.

**Electronic supplementary material:**

The online version of this article (10.1186/s12859-018-2074-9) contains supplementary material, which is available to authorized users.

## Background

Bioinformatics workflows typically consist of serially dependent operations including reading and parsing inputs, storing parsed data in memory as data structures, and computing on the stored data to generate desired outputs [1]. While individual steps could be accomplished manually using bioinformatics tools with graphic user interface (GUI) such as Galaxy [2], tools with command-line interface (CLI) are essential for automated processing. Take for example inference of bacterial phylogeny. It is desirable to compute a phylogenetic tree based on codon-based alignments rather than on directly aligned nucleotide sequences. There are two distinct approaches to develop a command-line pipeline for this purpose, both relying on biological Application Programming Interfaces (APIs) such as BioPerl and BioPython [3–5]. In one approach based on the BioPerl toolkit, one may compose a custom Perl script that calls the Bio::SeqIO module to read the nucleotide sequences and store them as Bio::Seq objects. Next, the nucleotide sequences will be translated into protein sequences, which are written out to a temporary file. The script will then call an external program (e.g., MUSCLE [6]) to align the protein sequences and produce a second temporary file, which will subsequently be read back and turned into a Bio::SimpleAlign object. Finally, the script will invoke the “aa_to_dna_aln()” method of the Bio::Align::Utilities module to produce the codon-based alignment using nucleotide sequences stored as Bio::Seq objects and the protein alignment stored as a Bio::SimpleAlign object. In a second approach, one may use existing (or design new) command-line utilities for each of the above steps and then accomplish the same task exclusively using commands on a Unix-like operating system, such as GNU/Linux or macOS.

The second, utility-based approach is preferable to the first, custom-script approach for the following reasons. First, Unix utilities are designed for serialized computation. In particular, by reading and writing on the standard streams and with the use of the pipe (“|”) operator, Unix utilities increase efficiency by keeping the rate-limiting step of reading and writing temporary files to a minimum. In the above example, both temporary files could be eliminated (see Advanced Usage (3) below). Second, bioinformatics applications built on utilities are more amenable for testing and results more reproducible. In our experience, it is far easier to maintain a code base of utilities that can be flexibly combined into robust pipelines than to maintain a repository of custom-made, single-use scripts. Third and more importantly, as a new computational layer between biological APIs and applications, bioinformatics utilities relieve bioinformaticians from the need to learn or to interact directly with the APIs. As such, the utility-based approach makes the APIs accessible to all users including non-programmers, meanwhile increasing the productivity of experienced users by allowing them to focus on computation and not on creating custom scripts prone to bugs and a short shelf life.

The enduring success of Unix-family text-parsing utilities illustrates the importance of well-designed command-line utilities for biological computing, meanwhile suggesting ways for designing such toolkits. Design of durable biological utilities would do well by following the so-called Unix Philosophy, with stipulations such as to “[M]ake each program do one thing well. Expect the output of every program to become the input to another, as yet unknown, program” [7]. EMBOSS (European Molecular Biology Open Software Suite, http://emboss.sourceforge.net/), a comprehensive collection of more than 100 command-line applications, is perhaps the most commonly used set of bioinformatics utilities [8]. The Newick Utilities (http://cegg.unige.ch/newick_utils) are a set of 18 command-line utilities for manipulation and visualization of phylogenetic trees [9]. More recently, the FAST (Fast Analysis of Sequences Toolbox, https://github.com/tlawrence3/FAST) suite explicitly follows the “pipes-and-filters” design principle of UNIX text utilities and currently consists of about more than 20 command-line utilities for manipulation of biological sequences [10].

Here we describe BpWrapper, a novel suite of command-line utilities for manipulating biological objects including sequences, alignments, and phylogenetic trees. Unlike EMBOSS and Newick Utilities but similar to FAST, BpWrapper utilities are built upon a robust and popular library of biological APIs with an active community of volunteer developers. BioPerl (http://bioperl.org), a part of the Open Bioinformatics Foundation (http://www.open-bio.org), is a pioneering and successful model for developing high-quality Open-Source software toolkits for life science applications [4, 5]. By wrapping BioPerl modules instead of programming from scratch, BpWrapper benefits from the continuous testing and development by the BioPerl community. Compared with EMBOSS, Newick Utilities, and FAST, BpWrapper improves usability by having a more concisely named user interface consisting of only four commands each with more extensive options, also in the tradition of Unix text-parsing utilities.

## Implementation & Results

### Basic usage

**Fig. 1:**
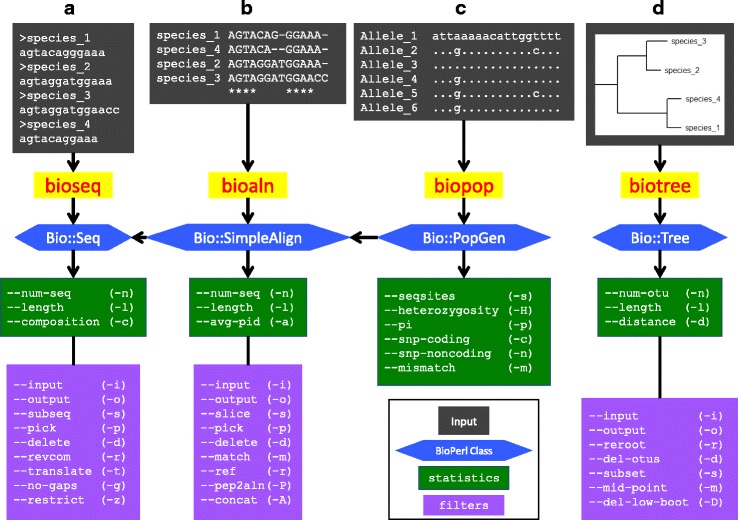
Four command-line utilities in BpWrapper. **a** bioseq reads sequences (in FASTA format as the default) as inputs, renders them into Bio::Seq objects in BioPerl (blue), and generates sequence statistics (green) or a modified FASTA file (purple). **b** bioaln reads a sequence alignment (in CLUSTALW format as the default) as input, renders them into a Bio::SimpleAlign object in BioPerl, and generates alignment statistics or a modified alignment. **c** biopop reads allelic sequences (in FASTA format as the default) as inputs, renders them into Bio::PopGen objects, and generates SNP (single-nucleotide polymorphism) statistics. **d** biotree reads a phylogenetic tree (in NEWICK format as the default) as inputs, renders it into a Bio::Tree::Tree object, and generates tree statistics or a modified tree. Note that since the Bio::PopGen class in BioPerl inherits the Bio::SimpleAlign class, which in turn inherits the Bio::Seq class, options in bioseq are applicable to alignments as well as to allelic sequences and options in bioaln are applicable to allelic sequence alignments. Documentation of these utilities are self-contained through the Perl POD mechanism and viewable on the command line through the “perldoc” command or the “--help”, “-h”, or “--man” options. A reference card of all options and their usage is provided in the Additional file 1

BpWrapper utilities are coded with Perl and BioPerl. The first release of BpWrapper consists of four utilities including bioseq, bioaln, biopop, and biotree for the processing of sequences, alignments, aligned allelic sequences, and phylogenetic trees, respectively (Fig. 1). Each utility reads a file (or from standard input) with a default file format and renders the file contents into an instance of a corresponding BioPerl class. Each option of the utilities either generates descript statistics of the object or outputs a transformed text stream onto standard output. The first three utilities could be used either independently or jointly due to class inheritance. For example, one could apply bioseq options to an alignment (but not vice versa). A selection of frequently used options and their basic usage are shown in Table 1. A complete list of options and their usages are available as embedded POD, accessible on the command line with the perldoc command or the “--help”, “-h”, or “--man” options.

**Table 1:** A selection of options and their usage

Utility	Option	Usage	Example
bioseq	--length, -l	Print lengths of sequences	bioseq –l foo.fasta
	--num-seq, -n	Print number of sequences	bioseq –n foo.fasta
	--composition, -c	Print base/residue composition	bioseq –c foo.fasta
	--revcom, -r	Reverse & complement	bioseq –r foo.fasta
	--pick, -p	Pick sequences by identifiers	bioseq –p ‘id:B31,N40’ foo.fasta
		Pick sequences by order	bioseq –p ‘order:1-3’ foo.fasta
		Pick sequences by pattern	bioseq –p ‘re:B31’ foo.fasta
	--delete, -d	Delete sequences by identifiers	bioseq –d ‘id:B31,N40’ foo.fasta
		Delete sequences by order	bioseq –d ‘order:1-3’ foo.fasta
		Delete sequences by pattern	bioseq –d ‘re:B31’ foo.fasta
	--subseq, -s	Get a sub-sequence	bioseq –s ‘10,20’ foo.fasta
	--translate, -t	Translate in the 1st reading frame	bioseq –t1 foo.fasta
		Translate in three reading frames	bioseq –t3 foo.fasta
		Translate in all six reading frames	bioseq –t6 foo.fasta
	--input, -i	Read a GenBank file	bioseq –i ‘genbank’ foo.gb
	--restrict	Print fragments by a restriction digest	bioseq –-restrict ‘EcoRI’ foo.fasta
bioaln	--length, -l	Print alignment length	bioaln –l foo.aln
	--num-seq, -n	Print number of sequences	bioaln –n foo.aln
	--avg-pid, -a	Print average percent identify	bioaln –a foo.aln
	--pick, -p	Pick sequences by identifiers	bioaln –p ‘id1, id2’ foo.aln
	--delete, -d	Delete sequences by identifiers	bioaln –d ‘id1, id2’ foo.aln
	--slice, -s	Slice an alignment	bioaln –s ‘10,20’ foo.aln
		Slice to the end	bioaln –s ‘20,-’ foo.aln
		Slice from the start	bioaln –s ‘-,20’ foo.aln
	--input, -i	Read a FASTA alignment	bioaln –I ‘fasta’ foo.fasta
	--output, -o	Write a PHYLIP alignment	bioaln –o ‘phylip’ foo.aln
	--concat, -A	Concatenate alignments	bioaln –A *.aln > concat.aln
	--pep2dna, -P	Generate a codon-based alignment	bioaln –P ‘cds.fas’ pep.aln > codon.aln
biopop	--segsites, -s	Print number of segregating sites	biopop –s pop.fasta
	--pi, -p	Print average nucleotide differences	biopop –p pop.fasta
	--mis-match, -m	Obtain pair-wise mismatch distribution	biopop –m pop.fasta
	--snp-coding, -c	Print coding SNP statistics	biopop –c pop.fasta
	--stats, -t	Print population statistics	biopop –t ‘pi,theta’ pop.fasta
biotree	--length, -l	Print total tree length	biotree –l foo.newick
	--mid-point, -m	Re-root at mid-point	biotree –m foo.newick
	--del-otus, -d	Delete OTUs by identifies	biotree –d ‘id1,id2’ foo.newick
	--subset, -s	Obtain a sub-tree of specified OTUs	biotree –s ‘id1,id2,id3,id4’ foo.newick
		Obtain a sub-tree from an internal node	biotree –s ‘node1’ foo.newick
	--reroot, -r	Re-root with a outgroup	biotree –r ‘otu1’ foo.newick
	--del-low-boot, -D	Delete low-support branches	biotree –D ‘75’ foo.newick
	--dist-all	Print pair-wise OTU distances	biotree –-dist-all foo.newick
	--as-text, -t	Preview tree in ASCII	biotree –t foo.newick

### Advanced usage

1. The following is a command-line pipeline to select a sequence (by matching the identifier containing the string “B31” using regular expression) from an alignment (“test-bioaln.aln” in the “test-files” directory), remove alignment gaps, and translate into an amino-acid sequence. It uses both bioaln and bioseq, to take advantage of inheritance of the Bio::SimpleAlign class from the Bio::Seq class:
  bioaln –o “fasta” test-files/test-bioaln.aln | bioseq –p “re:B31” | bioseq –g | bioseq –t1
2. The following piped commands cleans up an initial phylogenetic tree (“test-biotree.dnd” in the “test-files” directory) by rooting it at the mid-point, removing branches with less than 100% bootstrap support, and discarding two unwanted OTUs (identified as “B31” and “N40”). This pipeline was used to produce a phylogenetic tree of the PFam32 gene family in genomes of the Lyme disease pathogen *Borrelia burgdorferi* [11].
  biotree –m test-files/test-biotree.dnd | biotree –D “1.0” | biotree –d “B31,N40”
3. The following more sophisticated workflow uses three BpWrapper utilities and two external programs to produce a phylogenetic tree from a set of homologous protein-coding nucleotide sequences (“cds.fas” in the “test-files” directory). It first translates them into peptide sequences, which are then aligned with MUSCLE [6]. Subsequently, bioaln is called to generate the corresponding codon-based alignment, which are read by FastTree [12] to produce an approximate maximum-likelihood tree including estimates of branch support. Finally, biotree is called to generate a mid-point rooted tree with high bootstrap supports. The whole workflow is accomplished without generating a single temporary file or composing any custom shell script.
  bioseq –t1 cds.fas | muscle –clwstrict | bioaln --pep2dna “cds.fas” –o “fasta” | FastTree –nt | biotree –D “0.9” | biotree –m.

### Performance

We compared performance between BpWrapper and a selected set of sequence utilities by running the same task with the same input file and on the same computer system (with a AMD Opteron Quad-Core 2.1GHz Processor, Ubuntu Release 14.04 operating system, and 16GB physical memory). For example, we ran six-frame translation of an input file 100 times using the “bioseq -t6” command from BpWrapper and the “transeq –frame = 6” command from EMBOSS. The EMBOSS run was significantly faster than the BbWrapper run (mean system CPU time of 1.54 s for transeq and 9.41 s for bioseq, *p* = 2.5e-4 by *t*-test). Similarly, we calculated the depths of OTUs of a tree 100 times using the “biotree --depth” command from BbWrapper and the “nw --distance” utility from Newick Utilities. The Newick Utilities run was significantly faster than the BbWrapper run (mean system CPU time of 0.253 s for nw_distance and 1.33 s for biotree, *p* = 1.59e-2 by *t*-test). However, BbWrapper performs at similar levels as the FAST utilities. For example, we calculated sequence lengths 100 times using the “bioseq --length” command from BbWrapper and the “faslen” utility from FAST. The FAST Utilities run was slightly faster than the BbWrapper run (mean system CPU time of 1.63 s for faslen and 2.46 s for bioseq, *p* = 0.019 by *t*-test). These results are not surprising since EMBOSS and Newick Utilities are both pre-compiled binaries with no external dependency while BbWrapper and FAST both consist of Perl scripts compiled during runtime and with dependency on BioPerl.

### Testing & Support

We followed modern standard industry practice for software testing to assure proper functioning of BpWrapper. As the first tier of tests, our source code is hosted on a public repository Github under the BioPerl namespace (https://github.com/bioperl/p5-bpwrapper). Every time a change is committed to the repository, tests are run to assure that code still runs as expect. By this process of continuous Integration, we tested the code on every major release of Perl since 5.10 using an Ubuntu Virtual machine (provided by Travis CI). Further, we released BpWrapper on CPAN (https://metacpan.org/release/Bio-BPWrapper) to take advantage of efforts by volunteer testers who have downloaded the code from CPAN, run it, and made test results publically available (http://matrix.cpantesters.org/?dist=Bio-BPWrapper+1.11). This aspect is somewhat unique to the Perl community and allows CPAN code to be tested on a wider number and variety of versions of Perl than would otherwise be feasible with our own efforts. As a result, our code has been tested and run successfully on 86 configurations by the network of volunteer computers. Finally, when a user installs BbWrapper from CPAN using the cpan or cpanm command, our test scripts are run to make sure that the code runs in the user-specific computing environment. BpWrapper is in constant development and support is available by contacting the corresponding author.

## Discussion

While dependence on BioPerl confers BpWrapper with advantages including a robust development framework, continuous community support, and a longer life span, these benefits come with a cost of significantly reduced performance in comparison with pre-compiled utilities such as EMBOSS and Newick Utilities [8, 9]. Nevertheless, we routinely use BbWrapper to process a large amount of genome-scale data, e.g., concatenating ~ 2000 alignments and transforming trees with ~ 400 OTUs, without encountering excessive delay or code breakage.

Whereas existing sequence utilities (e.g., EMBOSS, Newick Utilities, and FAST) offer methods for manipulating sequences or Newick trees but not both, BpWrapper includes over a hundred methods for manipulating sequences, alignments, and phylogenetic trees, many of which are novel and not found in existing utilities. BpWrapper utilities are most similar to FAST utilities in design, implementation, and performance by virtual of their shared dependency on BioPerl. BpWrapper, however, offers dozens more new methods for manipulation of alignments than FAST, including, for example, bootstrapping an alignment (--bootstrap|-b), alignment concatenation (--concat|-A), obtaining a codon alignment based on aligned protein sequences (--pep2dna|-P), and various alignment permutations for evolutionary analyses (--shuffle-sites --mutate-sites). In addition, the command-line user interface of BpWrapper is simpler. FAST consists of 24 utilities named after Unix text utilities. We believe that the user interface of BpWrapper, with consolidated four utilities named after objects (sequence, alignment, population and tree), is more concise and more intuitive to end users.

From the onset, we took the “wrap-don’t-write” strategy in developing BbWrapper utilities to minimize the amount of independent code not vested by BioPerl. At current stage, however, many BbWrapper methods (e.g., picking and deleting sequences and OTUs) are custom routines that would be ideally merged into the corresponding upstream BioPerl classes. Future development of BbWrapper will also involve code optimization, additional methods, and scalable solutions.

Since BbWrapper hides the APIs from the end-users, it is not hard to envision a future when the four utilities are supported by APIs other than BioPerl, by a mixture of APIs, or by pre-compiled binaries. Indeed, as bioinformatics evolves, it is our hope that a universal and enduring set of commands (e.g., bioseq, bioaln, biopop, and biotree) and their associated options emerge for basic manipulations of sequences, alignments, and phylogenetic trees that outlast any particular operating systems or APIs, in the way that the concisely named and well-designed Unix text utilities (e.g., grep, cut, and sort) have outlived and prospered well beyond their original host operating systems.

## Conclusions

BpWrapper is a set of command-line utilities developed by wrapping upon some of the most commonly used BioPerl classes. Its user interface is designed by closely following the principles of Unix text utilities, including reading and writing on the standard streams, a concise namespace of main commands each specialized for a single type of biological object, and an extensible set of command options for object manipulations. With novel and up-to-date methods and by drastically reducing the need for composing one-off scripts, BpWrapper is suitable for rapid prototyping of bioinformatics pipelines for comparative genomics and other bioinformatics applications, to be used either alone or in conjunction with other sequence utilities.

## Additional file

Additional file 1: A reference card for the four BpWrapper utilities (PDF 758 kb)

## Abbreviations

API: Application Programming Interface
CPAN: Comprehensive Perl Archive Network
CPU: Central Processing Unit
EMBOSS: European Molecular Biology Open Software Suite
FAST: Fast Analysis of Sequences Toolbox
OTU: Operational Taxonomical Unit;
POD: Plain Old Document (a format for Perl embedded documentation)
