# Supplementary material for: BpWrapper: BioPerl-based sequence and tree utilities for rapid prototyping of bioinformatics pipelines
Source: BMC Bioinformatics. 2018 Mar 2;19:76. doi: 10.1186/s12859-018-2074-9 (PMC5833151; doi:10.1186/s12859-018-2074-9)
Supplement: Supplementary file 1 — A reference card for the four BpWrapper utilities (PDF 758 kb) [file 12859_2018_2074_MOESM1_ESM.pdf]

Help & Manual: -h | --help | --man | perldoc <cmd>

bioseq: Sequence Utility

FASTA descriptors

`-l | --length`	Length of sequences
`-n | --num-seq`	Number of sequences
`-c | --composition`	Base or aa composition

FASTA filter - Multiple sequences

`-r | --revcom`	Reverse-complement sequence
`-p | --pick 'tag:x'`	Pick seq by tag ("id", "order", or "regex")
`-d | --delete 'tag:x'`	Delete seq by tag ("id", "order", or "regex")
`-t | --translate 'n'`	Translate in 1,3 or 6 reading frames
`-g | --no-gaps`	Remove gaps

FASTA filter - Single sequence

`-s | --subseq 'x,y'`	Sub-sequence from positions *x* to *y* (inclusive)
`-R | --reloop 'x'`	re-circularize a bacterial genome at position *x*

Other options

`-B | --break`	Write a FASTA file for each sequence
`-C | --count-codons`	Count codons for sequence
`-F | --feat2fas`	Extract FASTA sequence from GenBank bacterial genome file
`-H | --hydroB`	Return Kyte-Doolittle hydropathicity (proteins)
`-G | --lead-gaps`	Count and return leading gaps
`-X | --remove-stop`	Remove stop codons
`-x | --restrict 'RE'`	Predict fragments from a restriction enzyme digestion
`--restrict-coord 'RE'`	Predict fragments from restriction enzyme digestion in BED format
`-o | --output 'format'`	Specify output file format. Default is "fasta". Optional format is "genbank"
`-i | --output 'format'`	Specify Input file format. Default is "fasta". Optional format is "genbank"
`-L | --linearize`	Linearize one sequence per line
`--split-cdhit`	Parse cdhit output .clstr file and generate a FASTA file for each CDHIT family

biotree: Tree Utility

`-i | --input 'format'`	Specify Input file format
`-l | --length`	Print total tree length
`-m | --mid-point`	Midpoint root a tree
`-u | --otus-num`	List all OTUs
`-d | --del-otus 'a,b,c'`	Delete OTUs
`--depth 'n1,n2,n3'`	Print depth to root for nodes
`--distance 'n1,n2'`	Distance between two nodes
`-D | --del-low-boot'0.9'`	Delete low-support (<0.9) branches
`-r | --reroot 'otu'`	Reroot with "otu" as outgroup
`-o | --output 'format'`	Output tree in "nhx" or "tabtree"
`-c |--ci 'trait-file'`	Consistency indices for binary traits
`-B | --clean-boot`	Remove branch support values
`-b | --clean-br`	Remove branch lengths
`--ead`	Edge-length abundance distribution
`--label-nodes`	Append IDs to all nodes
`--lca 'n1,n2,n3'`	Return ID of the last common ancestor
`-L | --length-all`	Print all nodes/branch length
`-ltt 'number_of_bins'`	Data from Lineage-through-time plot
`--multi2bi`	Multifurcating tree → bifurcating tree
`-U | --otus-desc 'n|all'`	Print all descendant OTUs of a node or all nodes
`--random 'n'`	Build tree of random subset of *n* OTUs
`--sis-pairs`	Print whether or not sisters for all pairs of OTUs
`-s | --subset 'otu1,otu2,otu3|innode'`	Build tree for specified OTUs or a clade defined by an internal node
`-t | --as-text`	Draw tree in ASCII text (for preview)
`--tree-shape`	Print input for R Package apTreeshape
`-w | --walk 'out'`	Print distances to all other OTUs from an OTU

biopop: PopGen Utility

`-s | --seg-sites`	Print number of segregating sites
`-p | --pi`	Print average pairwise nucleotide difference
`-f | --four-gametes`	Perform four-gamete tests for each SNP pair
`-c | --snp-coding`	Print SNP statistics for coding sequences
`-C | --snp-coding-long`	Print the above in long format
`-n | --snp-noncoding`	Print SNP statistics for coding or non-coding seqs
`-m | --mis-match`	Output data for mis-match distribution
`-b | --bi-sites`	Retain binary informative sites
`-H | --heterozygosity`	Print heterozygosity for each SNP site
`--bi-part`	Print binary Newick trees for all SNPs
`-b | --bi-sites`	Print alignment for binary-informative SNPs
`--bi-sites-for-r`	Print above to be read by R package "genetics"
`-t | --stats 'tag'`	Statistics ('pi', 'theda', 'tajima_d', per-site values)

bioaln: Alignment Utility

Alignment descriptors

`-l | --length`	Length of alignment
`-L | --list-ids`	List sequence IDs
`-n | --num-seq`	Number of aligned sequences
`-a | --avg-pid`	Average percent identity
`-w | --window 'n'`	Average difference by sliding window of size *n*.

Alignment viewers

`-c | --codon-view`	Codon view (in groups of 3 nucleotides)
`-m | --match`	Match view (highlight variable sites)

Alignment filters

`-d | --delete 's1,s2,s3'`	Delete sequence(s)
`-p | --pick 's1,s2,s3'`	Pick sequence(s)
`-i | --input 'format'`	Specify input format. ClutstalW is default.
`-o | --output 'format'`	Specify output format. ClutstalW is default.
`-g | --no-gaps`	Remove gapped sites
`-r | --ref-seq 'seq_id'`	Use *seq_id* as reference sequence
`-s | --slice 'x,y'`	Return an alignment slice from *x* to *y* (inclusive)
`-u | --uniq`	Remove redundant sequences
`-v | --var-sites`	Show only variable sites
`-P | --pep2dna 'cds.fas'`	Back align CDS to peptide alignment
`-D | --dna2pep`	DNA alignment to protein alignment

Evolutionary analysis

`-A | --concat *.aln`	Concatenate multiple alignments
`-B | --con-blocks 'n'`	Extract conserved blocks of size *n*
`-S | --shuffle_sites`	Make a column-permuted alignment
`-R | --resample 'n'`	Resample *n* aligned sequences
`-b | --boot`	Bootstrap an alignment
`-M | --permute-states`	Permute within columns (to test tree-ness)
`--remove-third`	Remove third site
`-I | --aln-index 'id,n'`	Return unaligned position for a sequence at *n*
`--binary`	Transform sequences into binary format
`--bin-inform`	Print only binary informative sites
`-C | --consensus 'n'`	Add an *n%* consensus sequences
`--gap-states, --gap-states2`	Print gap statistics per column
`-F | --no-flat`	Turns on `'begin-end'` naming
`--phy-nonint`	Generate non-interleaved PHYLIP output
`-E | --rm-col 'id'`	Remove columns with gap in sequence
`--select-third`	Generate alignment of every-third base
`--trim-ends`	Remove 5' and 3' gapped columns
`--upper`	Make uppercase alignment

BpWrapper Reference Card
© QiuLab • Hunter • CUNY (04/10/2017)
